# Exploring the evolution of biochemical models at the network level

**DOI:** 10.1371/journal.pone.0265735

**Published:** 2022-03-21

**Authors:** Tom Gebhardt, Vasundra Touré, Dagmar Waltemath, Olaf Wolkenhauer, Martin Scharm

**Affiliations:** 1 Department of Systems Biology and Bioinformatics, University of Rostock, Rostock, Germany; 2 Personalized Health Informatics Group, SIB Swiss Institute of Bioinformatics, Lausanne, Switzerland; 3 Medical Informatics Laboratory, Institute for Community Medicine, University Medicine Greifswald, Greifswald, Germany; 4 Leibniz-Institute for Food Systems Biology at the Technical University of Munich, Munich, Germany; ETH Zürich, SWITZERLAND

## Abstract

The evolution of biochemical models is difficult to track. At present, it is not possible to inspect the differences between model versions at the network level. Biochemical models are often constructed in a distributed, non-linear process: collaborators create model versions on different branches from novel information, model extensions, during curation and adaption. To discuss and align the versions, it is helpful to abstract the changes to the network level. The differences between two model versions can be detected by the software tool BiVeS. However, it cannot show the structural changes resulting from the differences. Here, we present a method to visualise the differences between model versions effectively. We developed a JSON schema to communicate the differences at the network level and extended BiVeS accordingly. Additionally, we developed DiVil, a web-based tool to represent the model and the differences as a standardised network using D3. It combines an automatic layout with an interactive user interface to improve the visualisation and to inspect the model. The network can be exported in standardised formats as images or markup language. Our method communicates the structural differences between model versions. It facilitates the discussion of changes and thus supports the collaborative and non-linear nature of model development.

**Availability and implementation:** DiVil prototype: https://divil.bio.informatik.uni-rostock.de, Code on GitHub: https://github.com/Gebbi8/DiVil, licensed under Apache License 2.0.

**Contact:** url="tom.gebhardt@uni-rostock.de.

## Introduction

**Fig 1:**
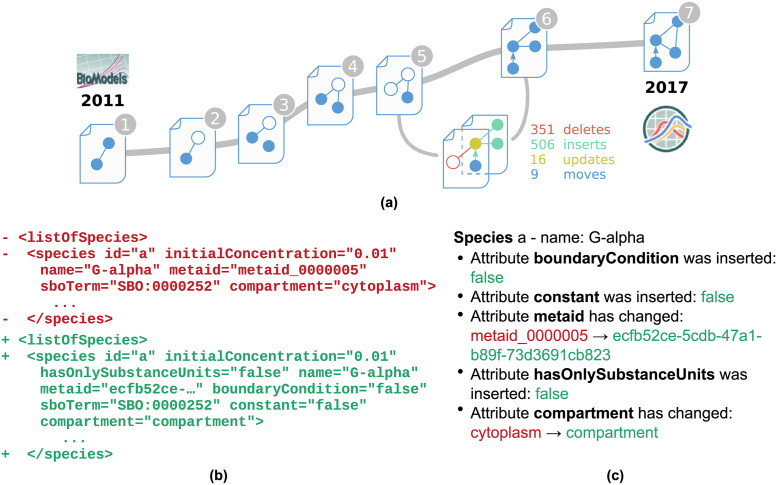
Abstract representation of the development of the Kummer2000 model [2]. Seven versions of the SBML model were published in BioModels between 2011 and 2017. Figure **(a)** shows the model’s evolution timeline. The slope between two versions represents the number of changes detected by BiVeS. When comparing the fifth and the sixth version a vast amount of changes are falsely detected by the default change detection tool Unix-Diff. The snippet from **(b)** shows that the Unix-Diff was not able to map species definitions on each other, although the compared species are very similar in both versions. Figure **(c)** shows a snippet of the BiVeS report for the same species. BiVeS maps both elements and correctly detects the changes in the attributes.

Models evolve over time. New insights, adaptions or extensions lead to several versions of the same model [1]. Furthermore, updates of the underlying model encoding formats may lead to changes in the syntactical representation of a model or result in different semantic information. One example is the *Oscillations in Calcium Signalling* model (Kummer2000) [2]. Seven versions of this model were published in the BioModels’ full releases (ftp.ebi.ac.uk/pub/databases/biomodels/releases/) between 2011 and 2017 [3]. A simplified view on the model’s evolution is shown in Fig 1a. The slope between two versions in the figure represents the amount of changes between them.

To date, the comparison of model files, in the frequently used formats SBML (Systems Biology Markup Language) [4] and CellML [5], is supported by the difference detection tool BiVeS. It exceeds default change detection systems, as used for Git [6] and SVN [7] repositories, by considering the formats’ formal languages [1].

Fig 1b depicts the differences between versions five and six of the *Kummer2000 model* as reported by Unix-Diff. The red lines (-) were detected as deletions, while the green lines are considered insertions (+). The snippet shows that the node *listOfSpecies* was deleted and also inserted. This tag is a mandatory and unique element of an SBML file. Thus, a mapping of both lines without a change would have been the correct detection. Furthermore, the snippet shows that a species was deleted in the fifth version and another species was added in the sixth version, while in fact only some attributes have changed.

Fig 1c shows part of a BiVeS output for the comparison of the same model versions and the same species. The algorithm implemented in BiVeS is domain-specific and hence understands parts of the semantics of SBML and CellML. BiVeS provides a correct mapping for the species, and it identifies the associated attribute changes [1]. Even though this is a tremendous improvement to the differences provided in Figure Fig 1b, it still demands effort to interpret the changes and mentally map them on the model’s network. An easy way to show the structural differences is missing.

Our novel method extends BiVeS and the visualisation library D3 [8] to enable difference visualisation of computational biology models encoded in SBML. We created a JSON schema that contains all mandatory and optional information to visualise differences between model versions. Our extension adapts BiVeS to the schema to export D3-readable JSON objects. We extended D3 to unambiguously visualise the differences with the System Biology Graphical Notation (SBGN) [9] standard. We added node shapes and arcs to enable D3 to depict SBGN Process Description [10] networks. Furthermore, we developed post production steps to reduce overlaps in the maps and hence improve the visualisation. With our method, the differences between two model versions can be viewed in a browser and exported in standard formats, such as SBGN-ML [11], SVG, and PNG. A prototype implementation of our method is openly available at: https://divil.bio.informatik.uni-rostock.de and has been put to use in https://most.bio.informatik.uni-rostock.de

## Materials and methods

Our method works with different standards for computational biology models. These standards are being maintained by the COmputational MOdeling in BIology NEtwork—COMBINE [12] to foster collaboration between researchers and to ensure interoperability of the developed standards and tools. Furthermore, we rely on the established difference detection tool BiVeS and frequently used visualisation library D3.

### Computational biology models

**Fig 2:**
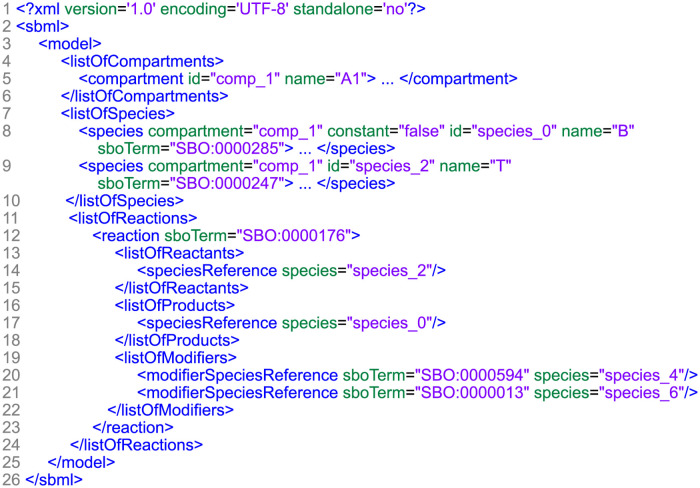
Condensed SBML file. This exemplary file was reduced to the information relevant to retrieve a standardised network visualisation. The information is encapsulated in the three lists: compartments, species and reactions.

Our work focuses on models encoded in the Systems Biology Markup Language (SBML). This XML-based format is regularly used in databases and publications to store biological models [13]. For instance, many SBML models are freely available in the BioModels database [3]. Fig 2 shows an SBML file that was condensed to all attributes and lists relevant for a standardised visualisation.

SBML uses specific types of data objects, organised as lists. Due to our structural interest in the model, the relevant information is enclosed in the lists of species, compartments and reactions. Species are the interacting elements of the model. They can have several attributes from which the unique identifier *id*, *compartment* and *sboTerm* are of major importance to us. The compartment attribute links to an object in the list of compartments through the use of a unique identifier. Compartments represent physical or functional groups of species, and are often used to represent an actual structure of the biological system, e.g. cells and organelles. The objects in the list of reactions represent processes that change the quantity of the species. This is not limited to interactions, but also represent transformation and transportation [13]. The participating species are linked in the contained lists of reactants, products and modifiers by their *id*. Reactions may have additional attributes in the reaction tag itself and in contained lists, of which *sboTerm* and *compartment* are of major interest to us. Each *sboTerm* contains a unique identifier representing a single concept of the Systems Biology Ontology [14]. *Compartment*s contain information about the location at which a reaction takes place.

### SBGN—Systems Biology Graphical Notation

The Systems Biology Graphical Notation (SBGN) is a community-driven standard developed for visualising biological networks in an unambiguous way. The notation defines symbols (called *glyphs*), for *species* that represent entities and for *arcs* that represent interactions [9]. Due to the existing possibilities to represent biological models and the different goals that their study can have, SBGN comes in three complementary languages:

- the Process Description (PD) [10] focusses on the mechanistic details of molecular interactions in a network,
- the Entity Relationship (ER) [15] focusses on every possible relationships of a given entity without considering temporal aspects, and
- the Activity Flow (AF) [16] shows the flow of information of activities that occurs between biochemical entities.

As we are interested in mechanistic models, we concentrate on SBGN PD.

### Systems Biology Ontology—SBO

As mentioned in section *Computational Biology Models* (page 3) models can be enriched with the *sboTerm* attribute. The Systems Biology Ontology (SBO) (https://www.ebi.ac.uk/sbo/main/) is a hierarchical structure of common terms which are used for the modelling of biochemical reaction networks [14]. Each SBGN glyph is mapped to an SBO term to disambiguate the role of the species and the type of processes. Models enriched with precise SBO terms make full use the abilities of SBGN. Therefore, we encourage all modellers to use proper SBO terms. In BioModels’ release from 2017, 322 of 640 curated models (≈50%) are annotated with SBO terms.

### Difference detection—BiVeS

The difference detection system BiVeS is a tool to identify the changes between two versions of a biochemical model. It outperforms the often used Unix-Diff by respecting the models’ hierarchical structure and ignoring properties that do not affect the model’s behaviour (e.g. white space, attribute reorder). Furthermore, additional post-processing steps are applied with respect to the model domain characteristics [1]. The BiVeS tool is available as a web service (https://bives.bio.informatik.uni-rostock.de/). It provides several human and machine readable outputs. The *report of changes* is text-based and simply lists the differences between the files which are relevant for the model. This output can be generated in the formats HTML, ReStructuredText and Markdown. Furthermore, BiVeS provides a *highlighted reaction network*. Therefor, an internal graph structure is computed which can be translated into the usual graph formats GraphML [17], Dot [18] and JSON [19]. The third type of output is a *delta encoded in XML*. It conveys all changes and allows for further computational processing of the retrieved information.

### Data Driven Documents—D3

**Fig 3:**
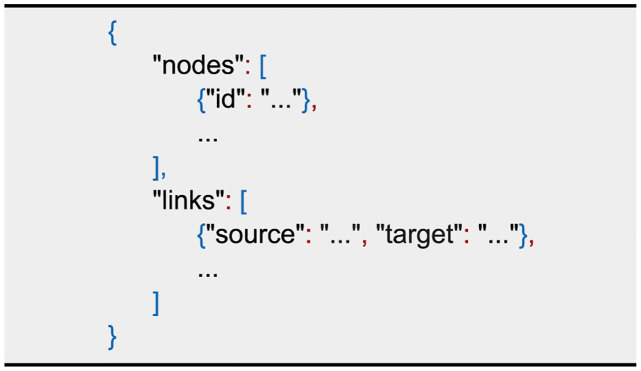
JSON input format for D3. The basic network level information are encapsulated in the lists nodes and links. Each element can be enriched with additional attributes.

D3 [8] is a JavaScript library for data visualisation which relies on the current web standards HTML5, SVG and CSS. It supports the visualisation of graphs, which is, in our case, an SBGN PD visualisation on an abstract level. Key features for our needs are a flexible input format, automatic layout, user interaction and an adjustable visualisation. Fig 3 shows the JSON object that D3 accepts as an input. It contains two lists, one for links and one for nodes, representing the edges and vertices of the graph. Since D3 can generate indices, no attribute is mandatory for a *node*. However, assigning unique identifiers makes it easier to access a specific node. A *link* always has information about the *source* and *target* which are referring to a node by its identifier. Both lists can be extended with additional attributes for each object to enhance the graph. D3 provides a force directed graph layout by calculating the velocity Verlet integration [20]. Several forces, such as node repulsion, spring forces by links and attraction to the canvas centre, can be applied to improve the layout and smooth the simulation. The tool provides the possibility to change the behaviour, look and position of the elements by user interaction or by modifying the nodes programmatically.

## Results

**Fig 4:**
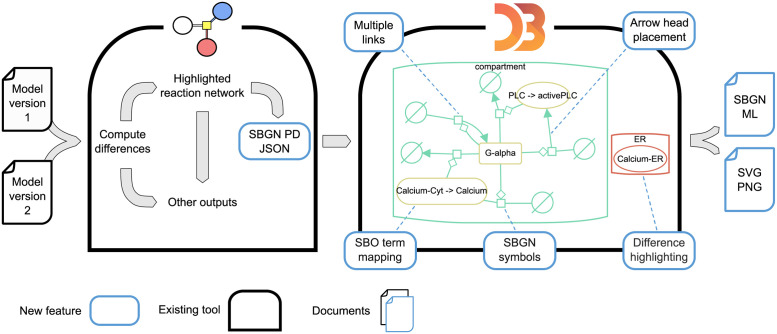
Workflow for the visualisation of differences between two model versions. BiVeS computes the differences between two model version files and provides several output formats to explore them. Based on BiVeS’ internal structure, we provide an additional output format to visualise the differences in D3. D3 needs several extensions to represent the network and differences in the standardised SBGN PD format. The right-hand side of the figure shows the result obtained from DiVil after comparing the Kummer2000 model versions five and six (see also Fig 1). We simplified the figure by deleting a few nodes to improve the readability. The coloured elements represent the changes detected by BiVeS. **Green** strokes indicate that the element was added in version six. **Red** elements occurred in version five but not six and **yellow** strokes indicate that the element occurred in both versions but attributes or sub nodes have changed.

We developed a method to visualise differences between versions of computational biology models based on the established tool *BiVeS* and the JavaScript library *D3*. Both tools are already broadly applied [21–24]. We connected the tools with a novel JSON schema and extended D3 with SBGN-compliant arcs and glyphs to create the difference visualisation tool **DiVil**. Several adjustments of D3’s automatic layout were necessary to avoid overlapping information and to visualise the differences. Fig 4 shows our workflow including the necessary extensions for difference visualisation.

### Connecting BiVeS and D3

**Fig 5:**
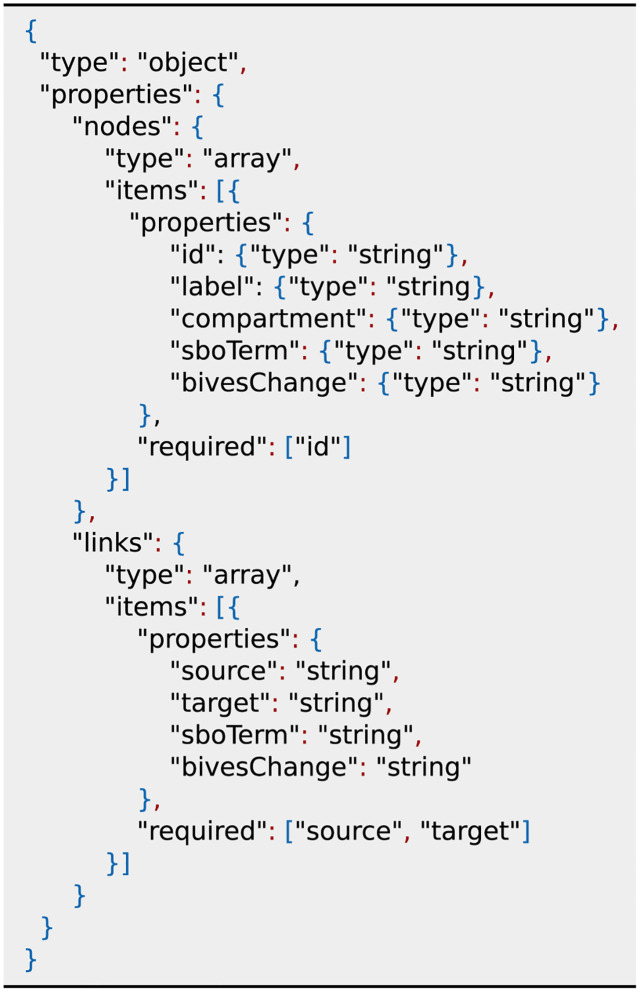
JSON schema to connect BiVeS and D3. Visualising differences in an SBGN PD network requires several information. D3 expects an array of nodes and links as input, representing the network structure. For nodes the attribute id is mandatory and links are required to have source and targets. These attributes are sufficient to build the network. Due to the default glyph and arc in SBGN, the other attributes are optional. However, we encourage everyone to make full use of the available node and link types to utilise the capabilities of SBGN PD and BiVeS.

We developed a JSON schema as an interface between BiVeS and D3. The main building blocks of this schema are the lists *nodes* and *links* which represent the network structure, as shown in Fig 5. A node either represents a species of the model or a process glyph from SBGN PD. Each node has a mandatory unique identifier *id*. A specific set of additional attributes can be added to transport all necessary information for a standardised visualisation of differences.

- **label** shown name of the node
- **compartment** id of a node containing the current node
- **sboTerm** links to an term from the Systems Biology Ontology
- **bivesChange** kind of detected change

A *link* shows the participation role of a species for a specific process. It carries the mandatory attributes *source* and *target*, which refer to node identifiers. Additionally, the attributes *class* and *bivesClass* can be used to communicate the SBO term and the detected change.

**Fig 6:**
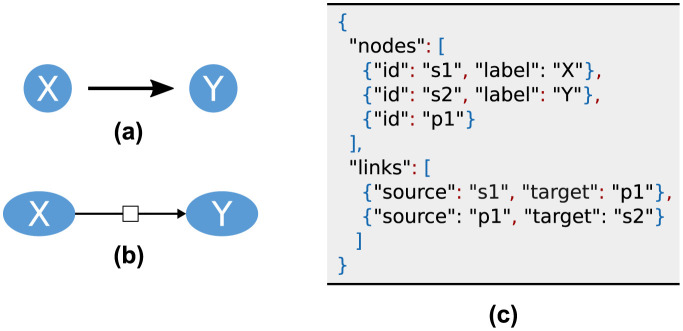
Mapping BiVeS’ internal structure to SBGN PD. Internally, BiVeS describes links as direct interactions between species **(a)**. In SBGN PD, every process requires a process node in which all links of this process start or end **(b)**. Thus, we add an additional node to our list of species and adapt the links accordingly. The translation from **(a)** to **(b)** is shown in Figure **(c)**.

We extended BiVeS according to our schema with the additional output format *SBGN PD JSON*. BiVeS describes links as direct interactions between species. As this is not SBGN PD conform, we add a node for each reaction of the model and adapt the links accordingly. Thus, a simple reaction from Species X to Species Y results in three nodes and two links in the JSON object as shown in Fig 6. The resulting output contains all species and processes from both model versions. The affiliation to the source document can be comprehended by the *bivesChange* attribute.

### SBGN PD as D3 visualisation

The basic set of symbols in D3 is not sufficient to visualise SBGN networks. Therefore, we constructed SBGN PD glyphs as scalable SVG paths to extend D3’s capabilities. The new symbols depend on a variable to set the size of the node while keeping a pleasant aspect ratio of the symbol itself and also between different symbols. The paths are mapped to the associated terms according to the SBGN PD specification. Furthermore, we added SBGN compliant arrow heads, which are also defined by SVG paths and appended as definitions to the browser’s SVG canvas. By referring to the definition the proper arrow head is set for each arc. The SBO terms of the species and modulators in the model files define which glyphs and arrow heads have to be used. Due to the hierarchical structure of SBO most glyphs and arcs have several associated terms. To our knowledge, an official mapping of the terms is currently missing. Thus, we created an JavaScript switch structure according to the hierarchy. The extension of D3 with SBGN PD notation and the translation of SBO terms is the basis for our difference visualisation tool **DiVil**. The following adaptions of D3 were mandatory to improve the readability and to enable the reuse of the visualisation.

### Placement of arrow heads

**Fig 7:**
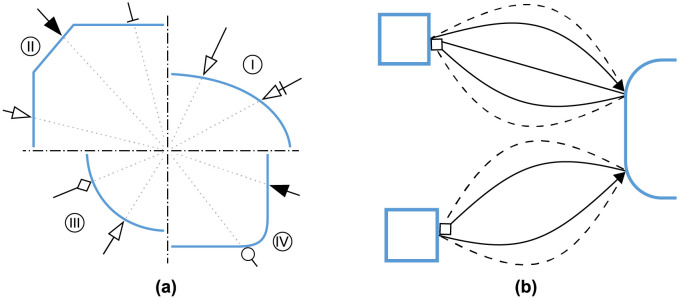
Geometric challenges when visualising SBGN PD networks. **(a)** Showcase of the arrow head placement for different arrow head and symbol combinations: **I** Unspecified entity, **II** Complex, **III** Simple Chemical and **IV** Macromolecule. **(b)** Visualising overlapping information. Showing the differences between two model version often results in several links between two nodes. By bending the link we are avoiding an overlap of the arcs. For each conflicting link the bend direction is altered, as shown on the bottom. Additionally we are increasing the bend for every 2*n* + 1 link. If the number of affected links is odd, as shown at the top, we are placing one arc as a straight link. The other links are computed as before.

The newly introduced SBGN glyphs have diverse shapes, while D3’s arrow head placement is based on a radius from the target nodes centre. To prevent an overlap of the nodes and arrow heads we calculate the crossing points between links and nodes and place the arrow heads accordingly. Some examples are shown in Fig 7a. For the calculation we split each glyph into logical parts depending on the positions of the target and the source node. The parts are then interpreted as their underlying geometric shape to compute the crossing points. Links without arrow heads are not part of this post processing to ease the computational effort. Our arrow head placement results in an appealing visualisation that improves the readability of the graph.

### Compartments around nodes

Compartments contain nodes and other compartments. Consequently, their placement depends on the position and size of their content. As a consequence, we first retrieve the compartment hierarchy and then start drawing the compartments in bottom-up order to prevent dependency issues. By considering all contained elements we are calculating the minimum and maximum coordinates as the boundary for each compartment. The calculation is chained at the end of D3’s automatic layout and updates after each layout step. Thus, the compartment visualisation appears smoothly during the force-directed layout and during user interaction.

### Interactive visualisation of differences

Since layout algorithms are limited and users may wish to to draw attention to specific nodes, we provide user interactions based on D3 functions. The nodes can be moved and fixed which triggers the automatic layout to adjust the other nodes. BiVeS distinguishes the detected differences in four types of change as explained in Section *Difference Detection—BiVeS* (page 4). We use these types as CSS classes to colour code the changes at the network level. Thus, the colours can be easily adapted, e.g. to different kinds of colourblindness, by adding a style document. In the prototype, as well as the example (Fig 4), we use the colour **red** to mark elements that only occurred in the first model version. **Green** strokes indicate, that the element was added in the second version. **Black** elements have been detected in both documents without a change. **Yellow** coloured nodes and links are elements that appeared in both versions but have changed attributes or sub nodes. While inserts and deletes are fairly easy to display and grasp, updates can convey several diverse changes. For instance, if a modulator was added to a reaction, the modulation arc would have a green stroke and the process node would be yellow. Additionally, other changes, e.g. in attributes values or local parameters, could affect the same node. To add more details about the visualised changes, we have implemented a popup overlay that lists the changes when a node is selected. The mapping of the changes is not straightforward, since our approach displays a network that includes both model versions. Thus, XML paths to the according document node can be ambiguous, due to moves. This kind of change has, so far, no influence on the network level but complicates the change mapping. Therefore, we compute a mapping that links every move to the path in the new document. With that, we can assign the changes accordingly. Depending on the type of change and the affected element, we display the changes in different formats to ensure good readability. Displaying kinetic laws is challenging, since SBML uses content MathML to encode the formula. We combined a script that converts the law into presentation MathML with MathJax [25] to reach a good visualisation.

### Multiple links between same nodes

The visualisation of differences between model versions often requires several links to be displayed between two nodes, which results in overlapping information. We therefore bend the nodes depending on the number of links as shown in Fig 7b. First we detect the overlapping links by comparing source and target nodes while also considering the reverse direction. We then use an SVG arc to visualise the link and set the bend based on the number of links and the direction. Due to the flexible network layout, the distance between nodes is not fixed. Thus, we chose arcs with a fixed bend. This approach avoids overlap and reduces edge crossing which highly improves the visualisation.

### Exporting the differences

Communication of the differences is elementary for collaborative model development. Thus, we provide an output of the visualisation in different formats. For static presentations a scale-able format (SVG) and a PNG file can be exported. Furthermore, we provide an output in the standardised format SBGN-ML. To do so, we convert the information of the browser’s SVG to provide the exact placement and size information of each node and link. By adding information from the JSON schema, we ensure to export the necessary data to visualise the same network in other tools with SBGN-ML input. Although other tools rarely interpret colours, we include the difference highlighting based on the render extension of the format. This will enable the reuse and adaption in interactive tools when the editors will evolve.

### Public instance of DiVil

**Fig 8:**
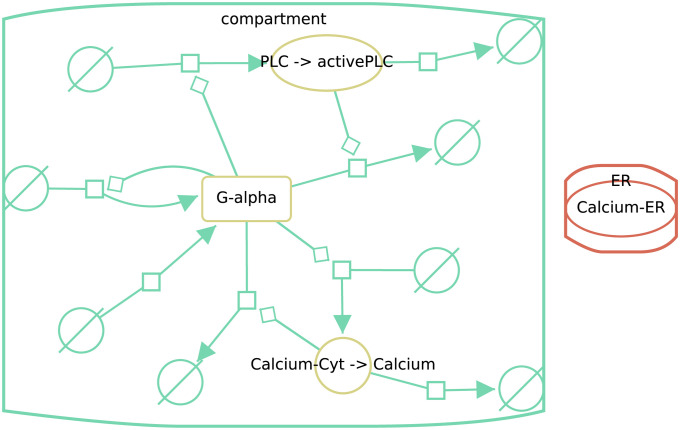
Screenshot of Divil’s output when comparing versions five and six of the Kummer2000 model, after manual layout improvements. Red strokes mark elements that only appeared in version five, green strokes indicate that the elements only occurred (and were added) in version six and yellow nodes have been updated during the version transition. In this example all reactions have been added, one compartment (ER) and one species (Calcium-ER) was deleted. Another compartment (compartment) was added, while three species where updated.

A public prototoype of DiVil (https://divil.bio.informatik.uni-rostock.de) is available, in which the capabilities of our approach can be explored. It works in all common browsers which adapt the ECMAScript [26] standard. At start, the differences between versions five and six of the Kummer2000 are displayed at the network level. With some user interaction the layout can be refined as observed in Fig 8. This example shows several changes. All processes have been added in version six which results in green process nodes, source and sink symbols and interaction links. The red stroke of the Clacium-ER node shows, that this element only occurred in version five and has been removed from version six. Furthermore, the red and green compartments display, respectively deletions and additions. When comparing the model files manually or with BiVeS, these obvious changes would be detectable.

**Fig 9:**
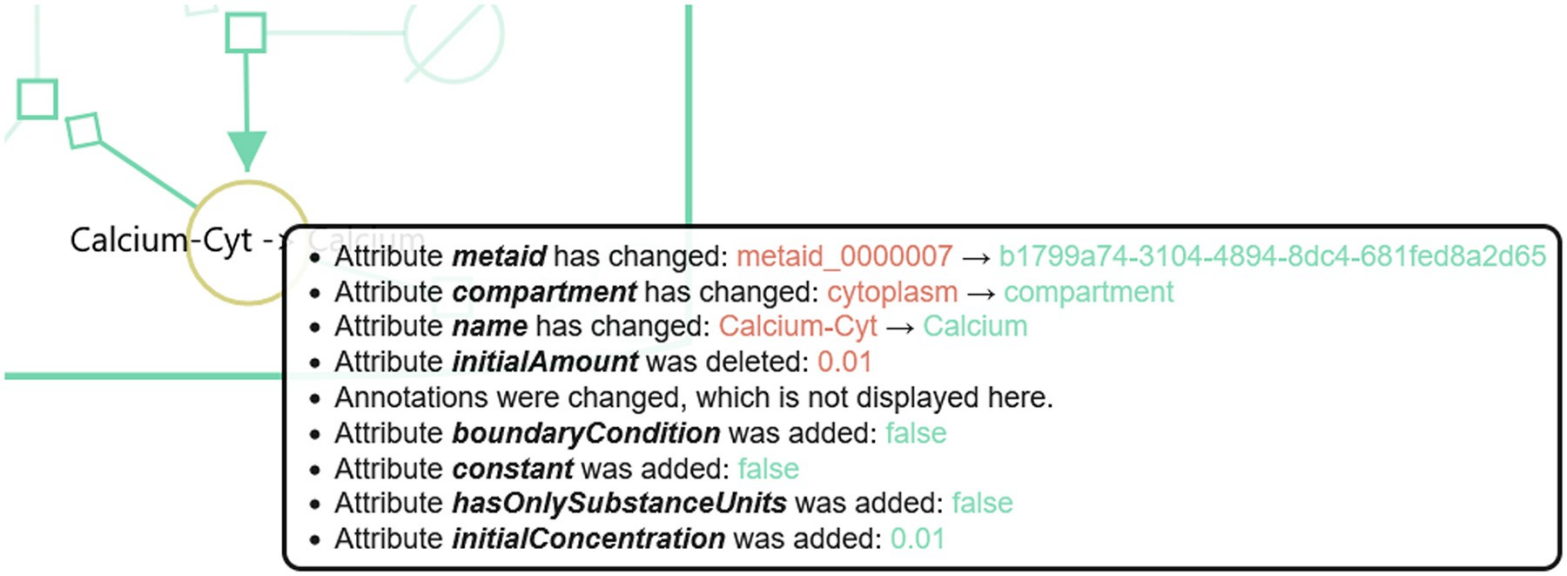
Change list for a species in DiVil. When comparing version five and six of the Kummer2000 model and selecting the Calcium species, a list of all relevant changes for this species is shown. Several attributes have changed during the version transition. E.g. the name of the species and its compartment were updated. Additionally attributes, such as initial concentration and constant were added. Changes in the annotations were also detected but not displayed in favour of a clear display.

Updates, which are marked in yellow, on the other hand are often hard to grasp. Most of the time they summarise multiple changes. Fig 9 shows the list of changes, displayed by DiVil, that led to the update of *Calcium-Cyt*. Some attributes, such as the name and the compartment have been updated. Others have been deleted or added.

**Fig 10:**
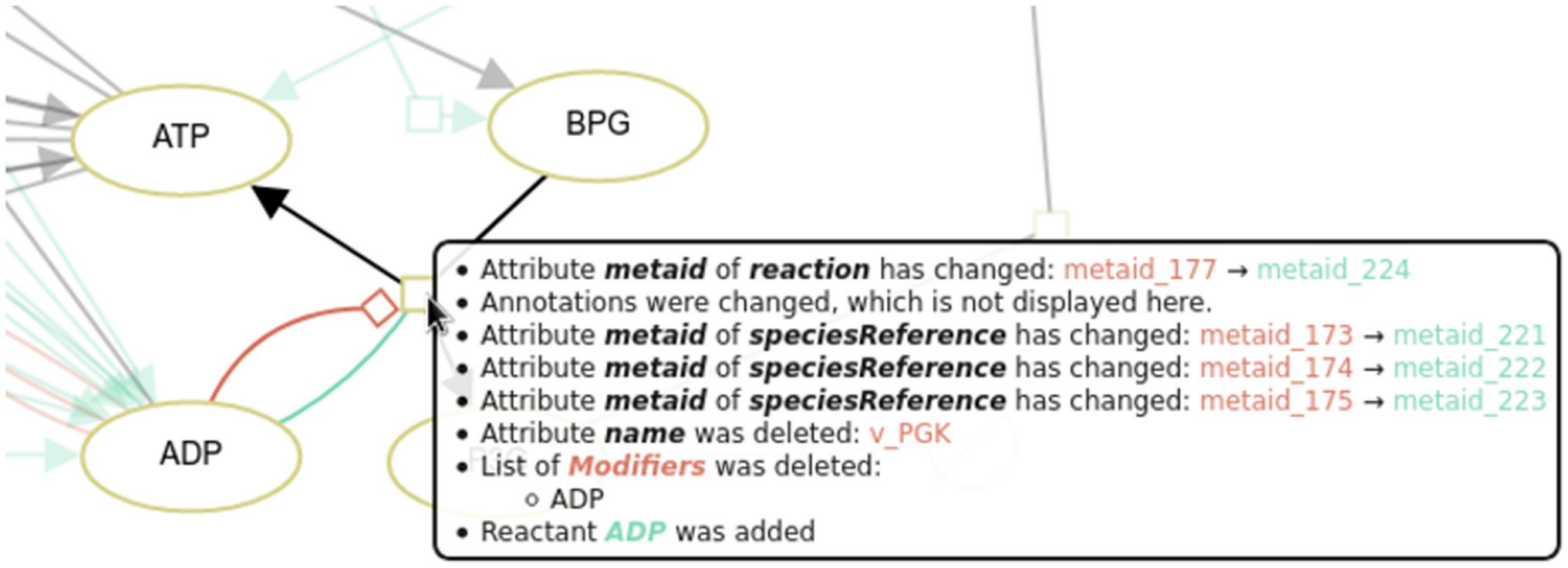
Change list for a reaction in DiVil. When comparing version six and seven of the Dupreez model several changes are detected for the reaction *ADP* + *BPG* → *ATP* + *P*3*G*. In version six ADP was a modifier in this reaction, while in version seven it is a reactant. Thus, in DiVil ADP is connected with a red modulation arc and a green consumption arc to the process node. Additionally, several meta ids of the reaction and its sub nodes were updated. The detected changes in annotations are not displayed in favour of a clear display.

Updated reactions can convey a wider range of changes. Updates of the participants and local parameters, e.g., would be mapped to the process node. Fig 10 shows a part of a screenshot in DiVil tool after comparing versions six and seven of the *Dupreez* model [27, 28] from JWS Online [29]. Several attributes have changed during the version transition. More importantly, the list of modifiers was deleted and the species ADP was added as a reactant. This results in a red modulation and a green consumption arc. After clicking on the process node, the changes can be perceived.

## Discussion

With our novel method we are able to unambiguously visualise the differences between versions of computational biology models at the network level. We connected the difference detection tool BiVeS and the visualisation library D3 with a JSON schema, introduced SBGN PD glyphs and arcs to D3, and improved the network layout. As input files we support SBML models.

We chose the **Kummer2000** model as the leading example for our paper, because it is large enough to have interesting changes but small enough to display them in a manuscript. Furthermore, it is properly annotated with SBO terms. However, the versions available at the BioModels FTP server are most likely not published by the research group, but are results of the ongoing curation process of the BioModels team. Nonetheless, it is interesting to apply our method to track the curation of the model. The perfect example would be versions of a model out of the development process, which are usually not published and not accessible.

Besides SBML, another frequently used standard for computational biology models is **CellML** [30] which is also XML-based and focuses on modelling of cellular functions. In contrast to SBML, CellML concentrates on expressing mathematical relationships of the underlying processes. Relations between entities on network is not mandatory and often missing. Therefore, we currently do not support CellML models.

According to the **SBGN PD** specification a process node has two ports: one connects the process to consumed elements (e.g. reactants), and the other one connects the process to products. This, however, constraints the layout and typically leads to additional edge crossings and overlaps. For example, reactants would need to be very close to each other to avoid an overlap of nodes and edges. This problem is amplified in force-based layouts: node positions depend primarily on their interaction partners. Thus, the species connected to a process node are often scattered and connecting the corresponding arcs to the same port without crossings is typically impossible. Ports ease the perception of reactants and products, but they do not provide additional information. Thus, we decided to neglect ports and trade minor SBGN PD compatibility for an enhanced visualisation. To compensate we take advantage of the interactive user interface and highlight interacting partners and connected arcs when hovering over a node.

**BiVeS** already provides a JSON format. It is suited to be visualised with Cytoscape.js [31], but it does not provide sufficient information for a standardised visualisation. Furthermore, the SBGN PD package available for Cytoscape does not support the visualisation of differences. Thus, we decided to develop the presented method.

DiVil is available as a **web application** from virtually any device and screen size. This, in turn, impacts its capabilities: a readable and interactive visualisation on small screens is limited by the number of represented nodes and links. Therefore, DiVil can be instantiated with a limited user interactions: on very small screens, such as smartphones, the graph layout is only computed for the first visualisation and cannot be modified by the user, which eases zooming on touch screens. However, selecting nodes to obtain further information and to highlight interaction networks remains possible.

Since differences are not considered in SBGN, exporting them in **SBGN-ML** is not yet straight forward. So far one can export colours, but they do not convey a specific meaning. Therefore, we added additional annotations from COMODI, an ontology specialised to characterise differences in computational models [32], to encode the meaning of the differences. This enables other tools to load and interpret the changes encoded in the graph.

The quality of a **network layout** is based on several attributes, such as the number of edge crosses and the length of the edges. Optimising these attributes is a well known challenge in the field of graph theory. Reducing the number of edge crosses is considered to be an NP-complete problem, meaning that it is unlikely to find an efficient algorithm [33]. An SBGN-PD visualisation implies additional layout goals, such as keeping nodes from the same compartment close to each other. This, in turn, magnifies DiVil’s challenge to generate a good network layout. Yet, by combining the automatic force based layout with an interactive user interface we enable the user to adjust the visualisation to their needs while keeping the effort for quick layouts low.

Due to the SBGN PD conform visualisation and the force based network layout the **applicability** of our method is **limited** by the combination of screen size, model size and computational effort. For large networks, such as disease maps [34, 35], the computational effort is too extensive but for average size networks the layout can be computed. Their readability depends on the number of links, which can lead to a cluttered visualisation. This approach finds its strength with smaller models since they are visualised fast and the changes are fairly easy to grasp in comparison to a report format.

We are confident that our method supports collaborative modelling and enables researchers to inspect changes in models intuitively. For future work we are pushing to support more steps of modern modelling in systems biology. Tracking the evolution of models during their development and enabling the merge of different development branches. Furthermore, we are aiming to integrate our tools in collaboration platforms.
